# The Effects of High Glucose on Adipogenic and Osteogenic Differentiation of Gestational Tissue-Derived MSCs

**DOI:** 10.1155/2016/9674614

**Published:** 2015-12-28

**Authors:** Weerawan Hankamolsiri, Sirikul Manochantr, Chairat Tantrawatpan, Duangrat Tantikanlayaporn, Pairath Tapanadechopone, Pakpoom Kheolamai

**Affiliations:** ^1^Medical Sciences Program (Cellular and Molecular Biology), Faculty of Medicine, Thammasat University, Pathumthani 12120, Thailand; ^2^Division of Cell Biology, Faculty of Medicine, Thammasat University, Pathumthani 12120, Thailand; ^3^Center of Excellence in Stem Cell Research, Thammasat University, Pathumthani 12120, Thailand; ^4^Department of Psychiatry, Faculty of Medicine, Thammasat University, Pathumthani 12120, Thailand

## Abstract

Most type 2 diabetic patients are obese who have increased number of visceral adipocytes. Those visceral adipocytes release several factors that enhance insulin resistance making diabetic treatment ineffective. It is known that significant percentages of visceral adipocytes are derived from mesenchymal stem cells and high glucose enhances adipogenic differentiation of mouse bone marrow-derived MSCs (BM-MSCs). However, the effect of high glucose on adipogenic differentiation of human bone marrow and gestational tissue-derived MSCs is still poorly characterized. This study aims to investigate the effects of high glucose on proliferation as well as adipogenic and osteogenic differentiation of human MSCs derived from bone marrow and several gestational tissues including chorion, placenta, and umbilical cord. We found that high glucose reduced proliferation but enhanced adipogenic differentiation of all MSCs examined. The expression levels of some adipogenic genes were also upregulated when MSCs were cultured in high glucose. Although high glucose transiently downregulated the expression levels of some osteogenic genes examined, its effect on the osteogenic differentiation levels of the MSCs is not clearly demonstrated. The knowledge gained from this study will increase our understanding about the effect of high glucose on adipogenic differentiation of MSCs and might lead to an improvement in the diabetic treatment in the future.

## 1. Introduction

Diabetes mellitus (DM) is a disease in which blood sugar level of patients is abnormally high. Diabetic patients are at risk of developing several disabling and life-threatening complications including cardiovascular diseases, cerebrovascular diseases, diabetic nephropathy, and diabetic retinopathy [1–3]. The important contributing factor for diabetic development is obesity which is defined as an excessive accumulation of body fat. Adipocytes in the accumulated fat tissues, especially around waist area and visceral organs, release several cytokines which reduce the sensitivity of several cell types to insulin leading to insulin resistant and type 2 diabetes [4, 5].

It is known that adipocytes are derived, at least in part, from multipotent mesenchymal stem cells (MSCs). Several recent studies demonstrated that high glucose reduced proliferation of rat bone marrow-derived MSCs (BM-MSCs) [6, 7] but enhanced adipogenic differentiation and lipid accumulation of mouse BM-MSCs [8], human osteosarcoma cell MG63 [9, 10] and human muscle-derived stem cells [11]. An increase in adipogenic differentiation levels was accompanied by an upregulation of the expression levels of several adipogenic genes including peroxisome proliferator-activated receptor gamma (*PPARγ*), adipocyte protein 2 (*AP2*), adipose tissue-specific secretory factor (*ADSF*), sterol regulatory element-binding protein 1C (*SREBP1C*), lipoprotein lipase (*LPL*), adiponectin (*ADIPOQ*), and glucose transporter type 4 (*GLUT4*) [8, 9, 11]. In contrast to adipogenic differentiation, high glucose suppressed osteogenic differentiation and downregulated the expression levels of osteogenic gene runt-related transcription factor 2 (*RUNX2*), collagen type I (*COL1A1*), osteonectin (*ON*), and osteocalcin (*OCN*) of mouse BM-MSCs and MG63 cells [7, 9].

Although the effects of high glucose on the proliferation and differentiation of rat and mouse BM-MSCs have been previously reported, the effects of high glucose on biological properties of human MSCs derived from gestational tissues which, due to their ease of isolation by noninvasive procedure, are considered more suitable sources of MSCs for clinical applications have yet to be determined. Therefore, the present study aims to investigate the effects of high glucose on proliferation and adipogenic and osteogenic differentiation of MSCs derived from bone marrow and several gestational tissues including chorion, placenta, and umbilical cord, as well as the mechanisms underlying those effects.

## 2. Materials and Methods

### 2.1. Subjects

This study was approved by the Institutional Review Board, Faculty of Medicine, Thammasat University, which was in accordance with the Declaration of Helsinki, the Belmont Report, CIOMS Guidelines, and ICH-GCP. Human bone marrow samples were obtained from healthy volunteers after giving written informed consents. The gestational tissues (umbilical cord, placenta, and chorion) were obtained from healthy newborns after receiving written informed consents from their mothers.

### 2.2. Isolation and Culture of MSCs

Mononuclear cells (MNCs) from human bone marrow were isolated using Ficoll-Hypaque (GE Healthcare, USA) density gradient centrifugation (2,000 rpm for 30 minutes at 20°C). Bone marrow-derived MNCs were then cultured in complete medium (Dulbecco's Modified Eagle's Medium (DMEM; Gibco BRL, USA) supplemented with 10% (v/v) fetal bovine serum (FBS; Gibco BRL, USA), 2 mM L-glutamine (Gibco BRL, USA), 100 U/mL penicillin, and 100 *μ*g/mL streptomycin (Gibco BRL, USA)) at the density of 1 × 10^6^ cells/cm^2^. Cultures were maintained at 37°C in a humidified atmosphere containing 5% CO_2_ and medium was replaced every 3 days throughout the entire culture period.

For the isolation of MSCs from umbilical cord, placenta, and chorion, the tissues were minced into small pieces and digested by incubating with 1.6 mg/mL collagenase XI (Sigma-Aldrich, USA) and 200 mg/mL deoxyribonuclease I (Sigma-Aldrich, USA) for 4 hours at 37°C with shaking. After incubation, the cells were washed twice with phosphate buffered saline (PBS; Gibco BRL, USA), resuspended in complete medium, and plated into 25 cm^2^ tissue culture flask (Corning, USA). Cultures were maintained at 37°C in a humidified atmosphere containing 5% CO_2_ and medium was replaced every 3 days throughout the entire culture period.

### 2.3. Immunophenotyping of MSCs

The 3rd–5th passage MSCs were characterized for their surface marker expressions by being incubated with the following mouse anti-human antibodies: anti-CD45-FITC (BioLegend, USA), anti-CD34-PE (BioLegend, USA), anti-CD90-FITC (BioLegend, USA), anti-CD73-PE (BioLegend, USA), and anti-CD105-PE (BD Pharmingen, USA) for 30 minutes at 4°C in the dark. After incubation, cell pellets were washed twice with PBS and fixed with 1% (w/v) paraformaldehyde in PBS. Flow cytometry was performed by FACScalibur flow cytometer (Becton Dickinson, USA) using CellQuest software (Becton Dickinson, USA).

### 2.4. Osteogenic and Adipogenic Differentiation of MSCs

The 3rd–5th passage MSCs were used to assess their adipogenic and osteogenic differentiation potential. For adipogenic differentiation, 7.5 × 10^4^ cells were seeded into an individual well of 6-well plate (Corning, USA) and cultured in adipogenic differentiation medium (complete medium supplemented with 0.1 *μ*M dexamethasone (Sigma-Aldrich, USA), 1 *μ*M insulin (Sigma-Aldrich, USA), 200 *μ*M indomethacin (Sigma-Aldrich, USA), and 250 *μ*M isobutylmethylxanthine (Sigma-Aldrich, USA)). Cultures were maintained at 37°C in a humidified atmosphere containing 5% CO_2_ and medium was replaced every 3 days throughout the entire culture period. After culture for 3 weeks, cells were fixed with formalin vapor, stained with 0.5% (w/v) oil red O (Sigma-Aldrich, USA) in isopropanol for 20 minutes at room temperature, and observed under light microscope (Nikon TS100, Japan) to determine number of MSC-derived adipocytes.

For osteogenic differentiation, 3 × 10^4^ cells were seeded into an individual well of 6-well plate (Corning, USA) and cultured in osteogenic differentiation medium (complete medium supplemented with 0.1 *μ*M dexamethasone (Sigma-Aldrich, USA), 50 *μ*g/mL L-ascorbic acid (Sigma-Aldrich, USA), and 10 mM *β*-glycerophosphate (Sigma-Aldrich, USA)). Cultures were maintained at 37°C in a humidified atmosphere containing 5% CO_2_ and medium was replaced every 3 days throughout the entire culture period. After culture for 3 weeks, cells were fixed with 4% paraformaldehyde and subjected to alkaline phosphatase activity assay using BCIP/NBT liquid substrate (Sigma-Aldrich, USA) to determine levels of osteogenic differentiation.

### 2.5. Effects of High Glucose on the Functional Properties of MSCs

#### 2.5.1. Effect on Proliferation

1 × 10^3^ 3rd–5th passage MSCs were seeded into an individual well of 24-well plate (Corning, USA) containing 500 *μ*L complete medium supplemented with 25 mM D-glucose which have been shown to be an optimal concentration of D-glucose for simulating high glucose condition by several previous studies [7–9, 11]. Cultures were maintained at 37°C in a humidified atmosphere containing 5% CO_2_ and medium was replaced every 3 days throughout the entire culture period. Cells were harvested on culture days 2, 4, 6, 8, 10, 12, and 14 to determine cell number. Dead cells were excluded by Trypan blue staining (Sigma-Aldrich, USA) and the number of viable cells was determined by hemocytometer. The average number of cells at each time point was then calculated and plotted against culture time to show growth kinetic. MSCs cultured in complete medium without D-glucose supplementation serve as controls.

#### 2.5.2. Effect on Adipogenic Differentiation

7.5 × 10^4^ 3rd–5th passage MSCs were seeded into an individual well of 6-well plates (Corning, USA) containing adipogenic differentiation medium supplemented with 25 mM D-glucose. Cultures were maintained at 37°C in a humidified atmosphere containing 5% CO_2_ and medium was replaced every 3 days throughout the entire culture period. The number of adipocyte-like cells was determined on culture day 14 (for BM-MSCs) and day 28 (for CH-MSCs, PL-MSCs, and UC-MSCs) by oil red O staining. To measure the amount of oil red O staining in each sample, oil red O presented in each stained sample was extracted by incubation with 1 mL isopropanol for 5 minutes at room temperature. The optical density (OD) of each extracted oil red O sample was measured by microplate reader (BioTex, USA) at the wavelength of 500 nm. The concentration of extracted oil red O in each sample was then calculated by comparing the measured OD values with a standard curve generated from various known concentrations of oil red O. MSCs cultured in adipogenic differentiation medium without D-glucose supplementation serve as controls.

#### 2.5.3. Effect on Osteogenic Differentiation

3 × 10^4^ 3rd–5th passage MSCs were seeded into an individual well of 6-well plates (Corning, USA) containing osteogenic differentiation medium supplemented with 25 mM D-glucose. Cultures were maintained at 37°C in a humidified atmosphere containing 5% CO_2_ and medium was replaced every 3 days throughout the entire culture period. After 14-day culture period, the levels of osteogenic differentiation were determined by alkaline phosphatase activity assay using BCIP/NBT liquid substrate (Sigma-Aldrich, USA) according to the manufacturer's instruction. MSCs cultured in osteogenic differentiation medium without D-glucose supplementation serve as controls.

#### 2.5.4. Effects on the Expression Levels of Adipogenic and Osteogenic Genes

To determine the effects of high glucose on the expression levels of adipogenic genes, 2 × 10^5^ 3rd–5th passage MSCs were cultured in 25 cm^2^ tissue culture flasks (Corning, USA) containing adipogenic differentiation medium supplemented with 25 mM D-glucose. On culture day 14 (for BM-MSCs) and culture day 28 (for CH-MSCs, PL-MSCs, and UC-MSCs), the expression levels of five adipogenic genes including* ADIPOQ*,* GLUT4*,* LPL*,* PPARγ*, and* SREBP1C* were determined by quantitative real-time PCR (qRT-PCR). MSCs cultured in adipogenic differentiation medium without D-glucose supplementation serve as controls.

To determine the effect of high glucose on the expression levels of osteogenic genes, 2 × 10^5^ 3rd–5th passage MSCs were cultured in 25 cm^2^ tissue culture flasks containing osteogenic differentiation medium supplemented with 25 mM D-glucose. On culture days 7 and 14, the expression levels of three osteogenic genes including* RUNX2*,* OSX*, and* OCN* were determined by qRT-PCR. MSCs cultured in osteogenic differentiation medium without D-glucose supplementation serve as controls.

### 2.6. Quantitative Real-Time PCR (qRT-PCR)

Total RNAs were isolated from cells using PureLink RNA Mini Kit (Invitrogen Corporation, USA) according to the manufacturer's instruction. cDNA was then synthesized from 2 *μ*g of RNA using SuperScript III Reverse Transcriptase (Invitrogen Corporation, USA). MicroAmp fast optical 96-well reaction plate (Applied Biosystem, USA) was used for qRT-PCR. Each well contained 3 *μ*L of cDNA, 1 *μ*L of 10 *μ*M forward and reverse primer mix, and 10 *μ*L of SYBR Green PCR Master Mix (Applied Biosystem, USA). Plates were sealed with MicroAmp clear adhesive film (Applied Biosystem, USA) to prevent evaporation of the reactant. PCR was performed using StepOne plus real-time PCR system (Applied Biosystem, USA) using the following protocol: 95°C initial denaturation for 10 minutes, followed by 40 cycles of denaturation (95°C, 10 seconds), annealing (60°C, 10 seconds), and extension (72°C, 40 seconds). Each sample was examined in duplicate and mean value was calculated. The quantitation was based on normalizing the level of gene of interest to the invariant control gene glyceraldehyde 3-phosphate dehydrogenase (*GAPDH*). Data were analyzed by comparative CT method using StepOne Software version 2.2 (Applied Biosystems; ABI, USA) and presented as relative mRNA levels. The primer sequences were listed in Table 1.

### 2.7. Statistical Analysis

Data were presented as mean ± standard error of the mean (SEM). Paired Student's *t*-tests were used to assess the significance of differences between observed data. *P* < 0.05 was considered to be statistically significant.

## 3. Result

### 3.1. Characteristics of Bone Marrow and Gestational Tissue-Derived MSCs

**Figure 1:**
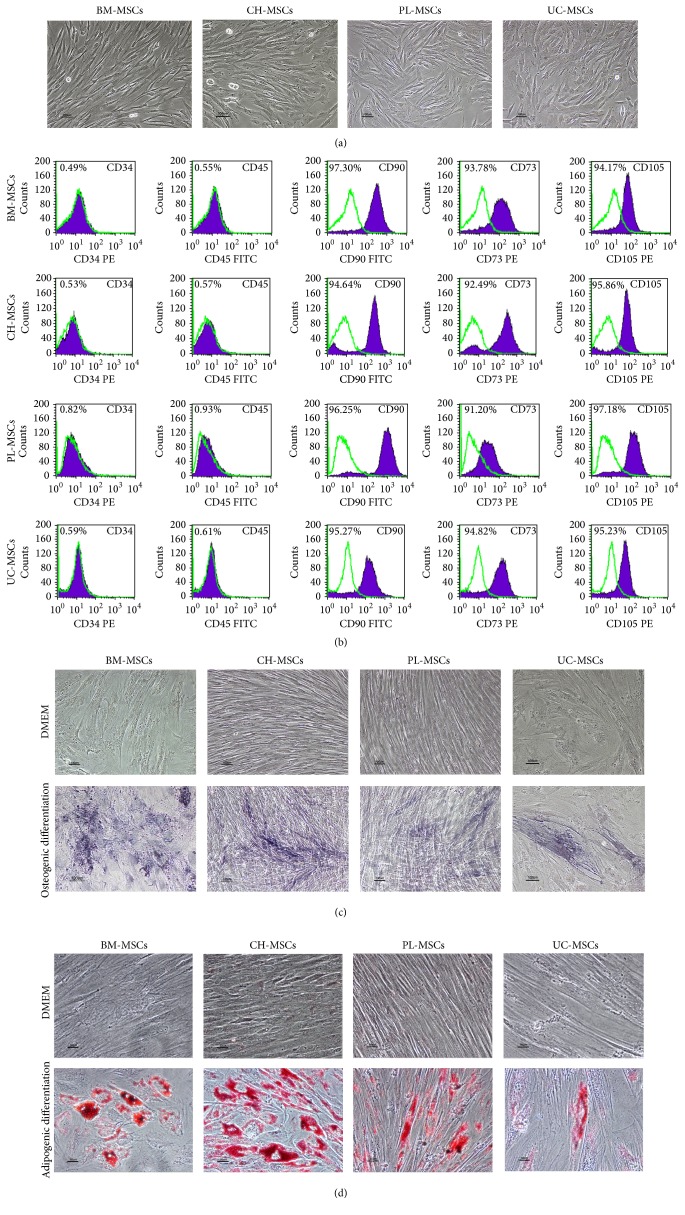
Characteristics of MSCs derived from bone marrow and gestational tissues. (a) Representative micrographs show the fibroblast-like morphology of BM-MSCs, CH-MSCs, PL-MSCs, and UC-MSCs. Scale bar: 100 *μ*m. (b) Immunophenotypes of BM-MSCs, CH-MSCs, PL-MSCs, and UC-MSCs as determined by flow cytometry. (c) Osteogenic differentiation of BM-MSCs, CH-MSCs, PL-MSCs, and UC-MSCs as determined by BCIP/NBT alkaline phosphatase activity assay. Scale bar: 100 *μ*m. (d) Adipogenic differentiation of BM-MSCs, CH-MSCs, PL-MSCs, and UC-MSCs as determined by oil red O staining. Scale bar: 50 *μ*m.

MSCs derived from gestational tissues including placenta (PL-MSCs), umbilical cord (UC-MSCs), and chorion (CH-MSCs) exhibited similar characteristics to that of bone marrow-derived MSCs (BM-MSCs). The gestational tissue-derived MSCs displayed fibroblast-like morphology (Figure 1(a)), expressed typical MSC surface markers (positive for CD73, CD90, CD105, and negative for hematopoietic markers CD34 and CD45; Figure 1(b)), and could differentiate to adipocytes and osteocytes as demonstrated by oil red O staining (Figure 1(c)) and alkaline phosphatase activity assay (Figure 1(d)), respectively. It is worth noting that CH-MSC, PL-MSCs, and UC-MSCs took a longer period of time to differentiate to adipocytes compared with BM-MSCs (28 days versus 14 days). Furthermore, the numbers of adipocyte-like cells generated from CH-MSC, PL-MSCs, and UC-MSCs were also lower than those generated from BM-MSCs.

### 3.2. Effect of High Glucose on MSC Proliferation

**Figure 2:**
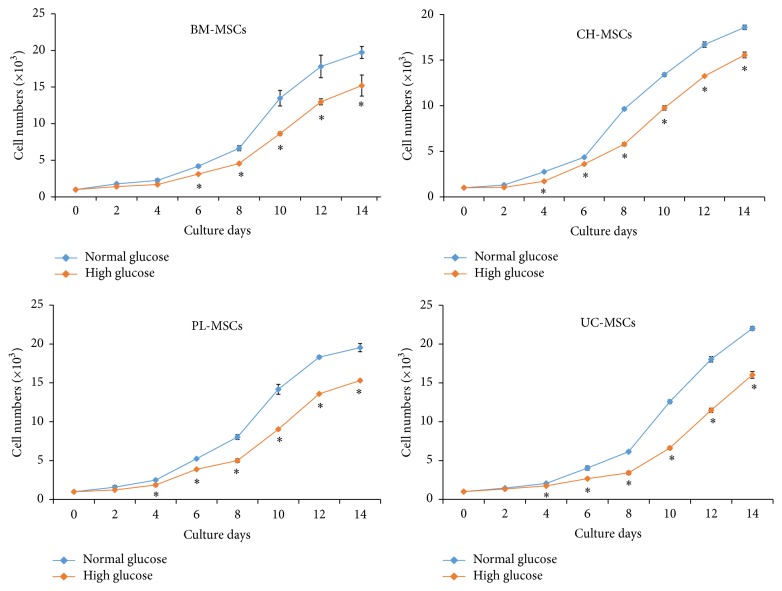
Effect of high glucose on MSC proliferation. Figure shows growth kinetic curve of BM-MSCs, CH-MSCs, PL-MSCs, and UC-MSCs during a 14-day culture period in high glucose condition (red lines). Data were presented as mean ± standard error of the mean (SEM) (*n* = 3). ^*∗*^ *P* < 0.05 versus normal glucose condition. MSCs cultured in complete medium without glucose supplementation (blue lines) serve as controls. *n* corresponds to the number of independent samples used in the experiments.

To study the effect of high glucose on MSC proliferation, BM-MSCs, CH-MSCs, UC-MSCs, and PL-MSCs were cultured in complete medium supplemented with 25 mM D-glucose for 14 days. The results showed that, from day 6 onward, the proliferation of BM-MSCs cultured in high glucose condition was significantly reduced when comparing with their normal glucose controls (Figure 2). Similar to BM-MSCs, the proliferation of CH-MSCs, PL-MSCs, and UC-MSCs cultured in high glucose condition was also significantly reduced when comparing with their normal glucose controls (Figure 2).

### 3.3. Effect of High Glucose on Adipogenic Differentiation of MSCs

**Figure 3:**
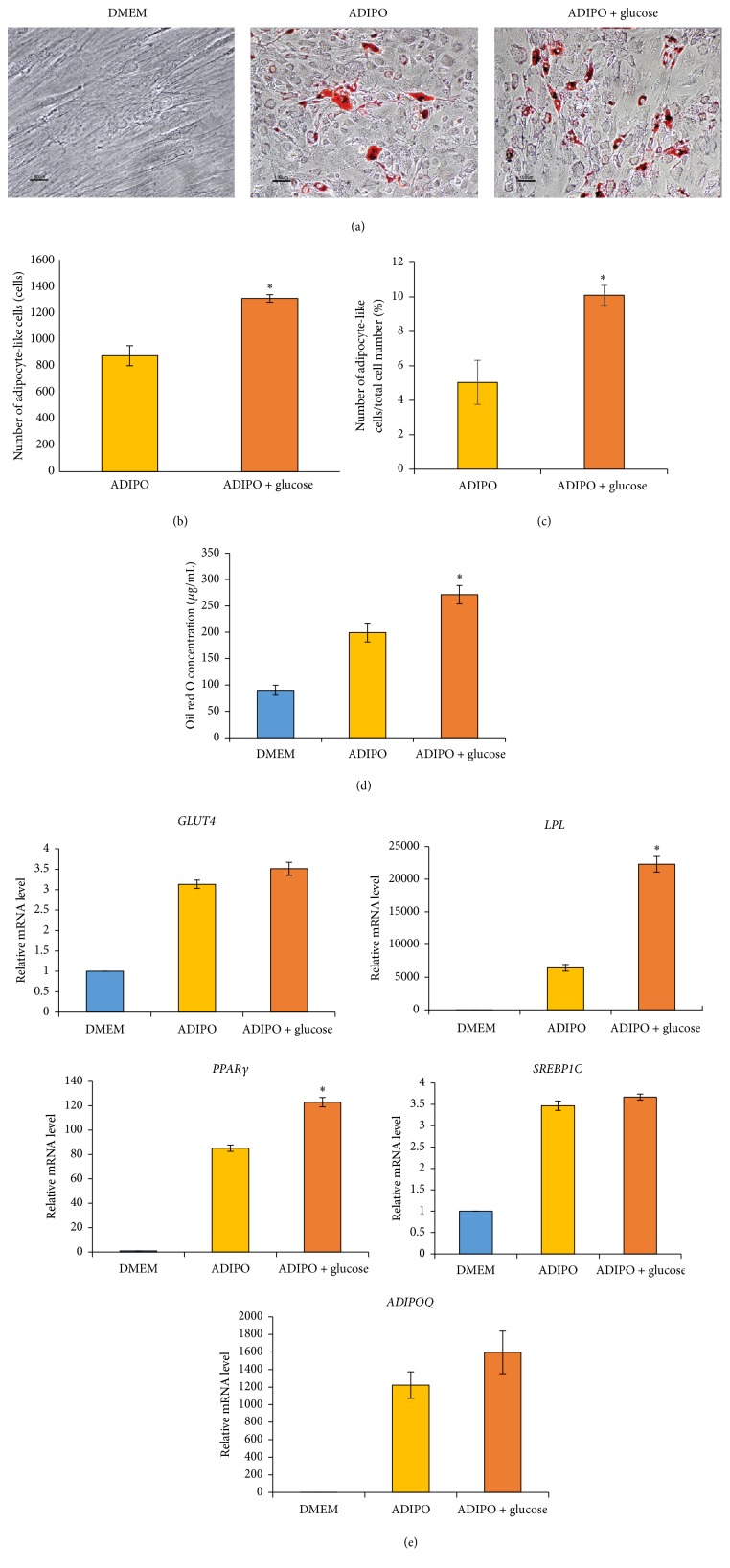
Effect of high glucose on adipogenic differentiation of BM-MSCs. (a) Representative micrographs show the morphology of adipocyte-like cells (red colored cells) derived from BM-MSCs cultured in adipogenic differentiation medium with or without glucose supplementation for 14 days after staining with oil red O. Scale bar: 50 *μ*m for DMEM, 100 *μ*m for ADIPO, and ADIPO + glucose. (b) Graph shows the number of adipocyte-like cells generated from BM-MSCs cultured in high glucose condition in comparison to their normal glucose controls on culture day 14 (*n* = 3). (c) Graph shows the percentages of adipocyte-like cells in total cell number generated from BM-MSCs cultured in high glucose condition in comparison to their normal glucose controls on culture day 14 (*n* = 3). (d) Graph shows the concentration of oil red O staining of BM-MSCs cultured in high glucose condition in comparison to their normal glucose controls on culture day 14 (*n* = 3). (e) Graph shows relative mRNA levels of adipogenic genes* ADIPOQ*,* GLUT4*,* LPL*,* PPARγ*, and* SREBP1C* of BM-MSCs cultured in high glucose condition in comparison to their normal glucose controls on culture day 14 (*n* = 3). Data were presented as mean ± standard error of the mean (SEM). ^*∗*^ *P* < 0.05 versus ADIPO. *n* corresponds to the number of independent samples used in the experiments. DMEM: BM-MSCs cultured in complete medium which serve as nondifferentiation controls. ADIPO: BM-MSCs cultured in adipogenic differentiation medium without glucose supplementation which serve as normal glucose controls. ADIPO + glucose: BM-MSCs cultured in adipogenic differentiation medium supplemented with 25 mM D-glucose.

To study the effect of high glucose on adipogenic differentiation of BM-MSCs, BM-MSCs were cultured in adipogenic differentiation medium supplemented with 25 mM D-glucose for 14 days. At the end of culture, the cells were stained with oil red O to determine number of adipocyte-like cells (Figure 3(a)). The results showed that the number of adipocyte-like cells and the percentages of adipocyte-like cells in total cell number derived from BM-MSCs cultured in high glucose condition were significantly greater than those derived from their normal glucose controls (1308.7 ± 28.8 versus 879.0 ± 75.7, ^*∗*^ *P* < 0.05, and 10.1 ± 0.6 versus 5.0 ± 1.3, ^*∗*^ *P* < 0.05, resp.) (Figures 3(b) and 3(c)). In agreement with these results, the concentration of oil red O extracted from BM-MSCs cultured in high glucose after oil red O staining was also significantly greater than that of their normal glucose controls (271.2 ± 17.5 versus 199.4 ± 18.0,  ^*∗*^ *P* < 0.05) (Figure 3(d)). In addition to the number of adipocyte-like cells, the mRNA levels of adipogenic genes* PPARγ* and* LPL* of BM-MSCs cultured in high glucose condition were also significantly upregulated when comparing with their normal glucose controls (Figure 3(e)). In contrast to* PPARγ* and* LPL*, the mRNA levels of other adipogenic genes including* SREBP1C*,* ADIPOQ*, and* GLUT4* of BM-MSCs cultured in high glucose were not different from their normal glucose controls (Figure 3(e)).

**Figure 4:**
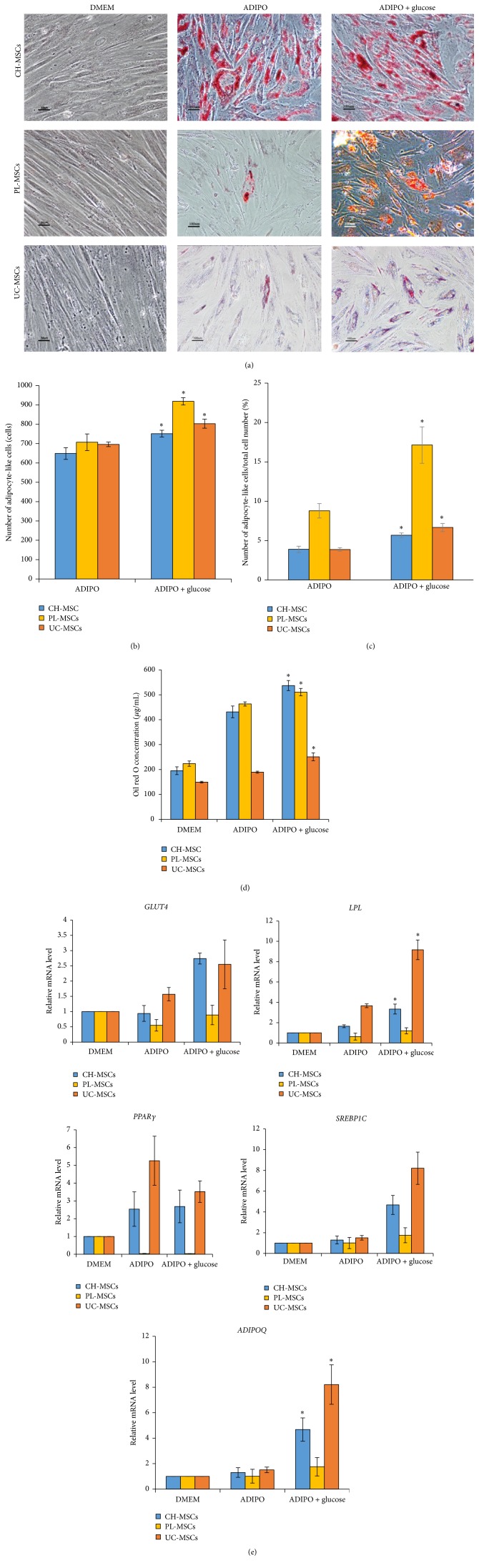
Effect of high glucose on adipogenic differentiation of gestational tissue-derived MSCs. (a) Representative micrographs show the morphology of adipocyte-like cells (red colored cells) derived from CH-MSCs, PL-MSCs, and UC-MSCs cultured in adipogenic differentiation medium with or without glucose supplementation for 28 days after staining with oil red O. Scale bar: 50 *μ*m for DMEM, 100 *μ*m for ADIPO, and ADIPO + glucose. (b) Graph shows the number of adipocyte-like cells generated from CH-MSCs, PL-MSCs, and UC-MSCs cultured in high glucose condition in comparison to their normal glucose controls on culture day 28 (*n* = 3). (c) Graph shows the percentages of adipocyte-like cells in total cell number generated from CH-MSCs, PL-MSCs, and UC-MSCs cultured in high glucose condition in comparison to their normal glucose controls on culture day 28 (*n* = 3). (d) Graph shows the concentrations of oil red O staining of CH-MSCs, PL-MSCs, and UC-MSCs cultured in high glucose condition in comparison to their normal glucose controls on culture day 28 (*n* = 3). (e) Graph shows relative mRNA levels of adipogenic genes* ADIPOQ*,* GLUT4*,* LPL*,* PPARγ*, and* SREBP1C* of CH-MSCs, PL-MSCs, and UC-MSCs cultured in high glucose condition in comparison to their normal glucose controls on culture day 28 (*n* = 3). Data were presented as mean ± standard error of the mean (SEM). ^*∗*^ *P* < 0.05 versus ADIPO. *n* corresponds to the number of independent samples used in the experiments. DMEM: MSCs cultured in complete medium which serve as nondifferentiation controls. ADIPO: MSCs cultured in adipogenic differentiation medium without glucose supplementation which serve as normal glucose controls. ADIPO + glucose: MSCs cultured in adipogenic differentiation medium supplemented with 25 mM D-glucose.

Similar to BM-MSCs, the numbers of adipocyte-like cells derived from CH-MSCs, PL-MSCs, and UC-MSCs cultured in high glucose condition were significantly greater than those derived from their normal glucose controls [751.7 ± 17.6 versus 639.0 ± 30.2, ^*∗*^ *P* < 0.05 for CH-MSCs; 919.0 ± 18.9 versus 707.3 ± 42.8, ^*∗*^ *P* < 0.05 for PL-MSCs; and 803.3 ± 23.3 versus 698.3 ± 11.7, ^*∗*^ *P* < 0.05 for UC-MSCs] (Figures 4(a) and 4(b)). The percentages of adipocyte-like cells in total cell number derived from CH-MSCs, PL-MSCs, and UC-MSCs cultured in high glucose condition were also significantly greater than those derived from their normal glucose controls [5.7 ± 0.3 versus 3.9 ± 0.4, ^*∗*^ *P* < 0.05 for CH-MSCs; 17.1 ± 2.3 versus 8.8 ± 0.9, ^*∗*^ *P* < 0.05 for PL-MSCs; and 6.7 ± 0.5 versus 3.9 ± 0.2, ^*∗*^ *P* < 0.05 for UC-MSCs] (Figure 4(c)). In agreement with these results, the concentrations of oil red O extracted from CH-MSCs, PL-MSCs, and UC-MSCs cultured in high glucose condition after oil red O staining were also significantly greater than those of their normal glucose controls [537.1 ± 20.0 versus 431.5 ± 23.6, ^*∗*^ *P* < 0.05 for CH-MSCs; 510.7 ± 15.6 versus 463.2 ± 4.0, ^*∗*^ *P* < 0.05 for PL-MSCs; and 251.1 ± 14.8 versus 189.4 ± 8.4, ^*∗*^ *P* < 0.05 for UC-MSCs] (Figure 4(d)). In addition to the number of adipocyte-like cells, the mRNA levels of adipogenic genes* ADIPOQ* and* LPL* of CH-MSCs and UC-MSCs cultured in high glucose condition were also upregulated when comparing with their normal glucose controls (Figure 4(e)). In contrast to* ADIPOQ* and* LPL*, the mRNA levels of other adipogenic genes including* PPARγ*,* SREBP1C*, and* GLUT4* in CH-MSCs and UC-MSCs cultured in high glucose were not different from those of their normal glucose controls (Figure 4(e)).

### 3.4. Effect of High Glucose on Osteogenic Differentiation of MSCs

To study the effect of high glucose on osteogenic differentiation, BM-MSCs, CH-MSCs, PL-MSCs, and UC-MSCs were cultured in osteogenic differentiation medium supplemented with 25 mM D-glucose for 14 days. On culture days 7 and 14, the expression levels of osteogenic genes* RUNX2*,* OSX*, and* OCN* were determined by qRT-PCR. The cultures were also subjected to alkaline phosphatase activity assay on culture day 14 to determine levels of osteogenic differentiation.

**Figure 5:**
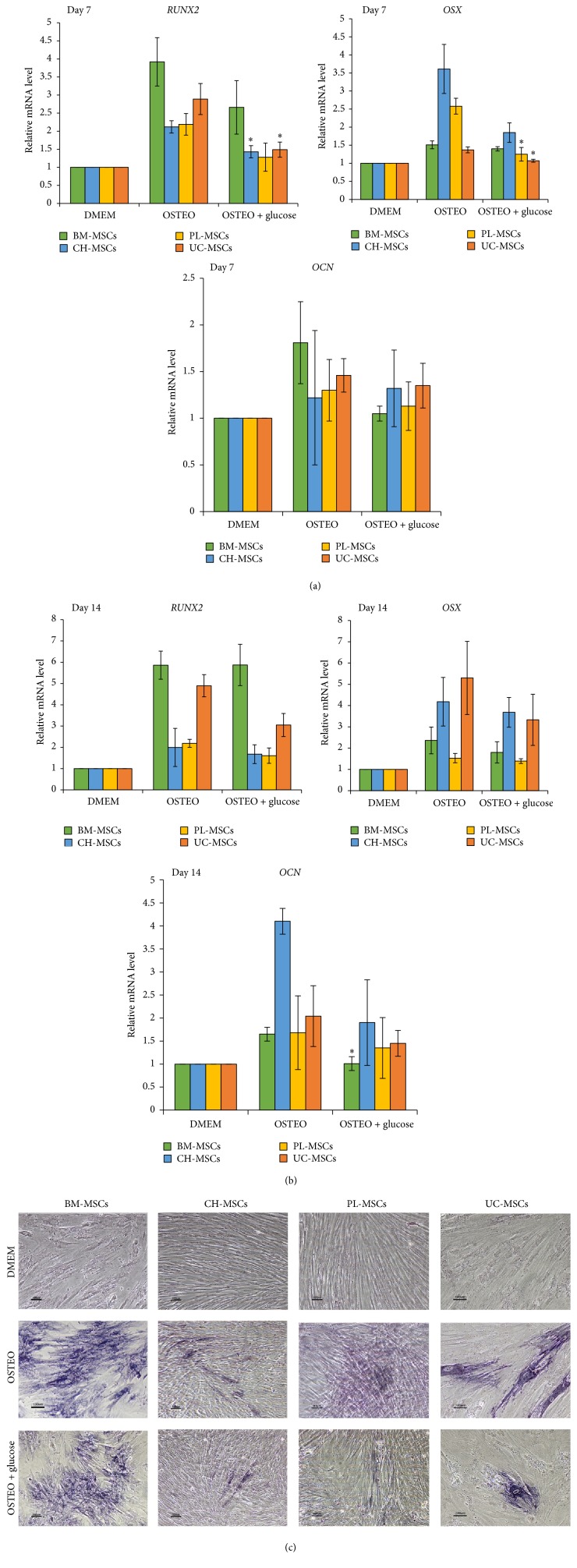
The effect of high glucose on osteogenic differentiation of BM-MSCs, CH-MSCs, PL-MSCs, and UC-MSCs. (a) Graph shows relative mRNA levels of osteogenic genes* RUNX2*,* OSX*, and* OCN* of BM-MSCs, CH-MSCs, PL-MSCs, and UC-MSCs cultured in high glucose condition in comparison to their normal glucose controls on culture day 7 (*n* = 3). (b) Graph shows relative mRNA levels of osteogenic genes* RUNX2*,* OSX*, and* OCN* of BM-MSCs, CH-MSCs, PL-MSCs, and UC-MSCs cultured in high glucose condition in comparison to their normal glucose controls on culture day 14 (*n* = 3). (c) Representative micrographs show the morphology of osteocyte-like cells (purple colored cells) derived from BM-MSCs, CH-MSCs, PL-MSCs, and UC-MSCs cultured in osteogenic differentiation medium with or without glucose supplementation for 14 days after being subjected to BCIP/NBT alkaline phosphatase activity assay. Scale bar: 100 *μ*m. Data were presented as mean ± standard error of the mean (SEM). ^*∗*^ *P* < 0.05 versus OSTEO. *n* corresponds to the number of independent samples used in the experiments. DMEM: MSCs cultured in complete medium which serve as nondifferentiation controls. OSTEO: MSCs cultured in osteogenic differentiation medium without glucose supplementation which serve as normal glucose controls. OSTEO + glucose: MSCs cultured in osteogenic differentiation medium supplemented with 25 mM D-glucose.

Although mRNA levels of* RUNX2* and* OSX* in CH-MSCs, PL-MSCs, and UC-MSCs cultured in high glucose were significantly lower than those of their normal glucose controls on culture day 7 (Figure 5(a)), the differences in mRNA levels of those genes could not be detected on culture day 14 (Figure 5(b)). In contrast to* RUNX2* and* OSX*, the mRNA levels of* OCN* in CH-MSCs, PL-MSCs, and UC-MSCs cultured in high glucose condition were not different from those of their normal glucose controls at both time points (Figures 5(a) and 5(b)). In case of BM-MSCs, there was no difference in the mRNA levels of all osteogenic genes examined at both time points, with the exception of* OCN* in which its mRNA levels of BM-MSCs cultured in high glucose condition were slightly lower than that of its normal glucose control on culture day 14 (Figure 5(b)).

In agreement with the gene expression study, alkaline phosphatase activity assay showed that the levels of osteogenic differentiation of BM-MSCs, CH-MSCs, PL-MSCs, and UC-MSCs in high glucose condition as determined by the alkaline phosphatase activity assay were not obviously different from their normal glucose controls (Figure 5(c)).

## 4. Discussion

Type 2 diabetic patients have increased risks of developing serious long-term complications, such as cardiovascular disease, retinopathy, nephropathy, neuropathy, diabetic foot, and osteoporosis, making diabetes a serious global health problem [1–3]. Most type 2 diabetic patients are obese who have an excessive visceral fat accumulated in their abdominal area. Adipocytes presented in the excessive visceral fat release several factors including fatty acids, glycerol, hormones, and proinflammatory cytokines that increase insulin resistance making diabetic treatment ineffective [12].

It is known that adipocytes, which are the major components of fat tissues, are derived, at least in part, from mesenchymal stem cells [13]. Although the effects of high glucose on the proliferation and adipogenic and osteogenic differentiation of mouse and rat BM-MSCs have been previously reported [6, 8, 9], the effects of high glucose on the properties of gestational tissue-derived MSCs especially on their adipogenic and osteogenic differentiation are currently unknown.

In the present study, we successfully isolated MSCs from several gestational tissues including CH-MSCs, PL-MSCs, and UC-MSCs. Similar to BM-MSCs, these gestational tissue-derived MSCs displayed fibroblast-like morphology, exhibited typical MSC surface markers, and could differentiate to osteocytes and adipocytes. The characteristics of CH-MSCs, PL-MSCs, and UC-MSCs established in this study were similar to those described in the previous reports [14–19]. Our observation showed that CH-MSCs, PL-MSCs, and UC-MSCs took a longer period of time than BM-MSCs to differentiate to adipocytes and osteocytes and the numbers of adipocytes and osteocytes derived from these MSC sources were lower than those derived from BM-MSCs cultured under the same conditions. These results reflect the endogenous differences in the osteogenic and adipogenic differentiation capacity between gestational tissue-derived MSCs and BM-MSCs.

Similar to BM-MSCs, high glucose suppressed the proliferation of CH-MSCs, PL-MSCs, and UC-MSCs. This result is in agreement with previous reports showing that high glucose inhibited proliferation of mouse BM-MSCs [7, 8] and human osteosarcoma MG63 cells [9, 10] by modulating JAK/STAT and p38 signaling pathways [20, 21].

Although previous studies demonstrated that high glucose enhanced adipogenic differentiation of mouse BM-MSCs [7, 8], MG63 cell [9], and human muscle-derived stem cells [11] by upregulating the expression of several adipogenic genes [8, 9, 11], the effects of high glucose on adipogenic differentiation of gestational tissue-derived MSCs have not yet been determined. This study demonstrates for the first time that high glucose induced the expression of adipogenic gene* PPARγ* and* LPL* in BM-MSCs, as well as* ADIPOQ* and* LPL* in CH-MSCs, PL-MSCs, and UC-MSCs. The upregulation of these key regulators of adipogenesis enhanced adipogenic differentiation of BM-MSCs, CH-MSCs, PL-MSCs, and UC-MSCs as demonstrated by an increase of both adipocyte number and extent of oil red O staining in MSCs cultured in high glucose condition. These results are in agreement with the previous studies which reported that high glucose upregulated the expression of several adipogenic genes including* PPARγ*,* SREBP1C*,* ADIPOQ*,* LPL*, and* GLUT4* in mouse BM-MSCs, MG63 cells and human muscle-derived stem cells [8, 9, 11].

We observed both similarity and variability in adipogenic gene expression between bone marrow- and gestational tissue-derived MSCs which were cultured in high glucose condition. While high glucose clearly upregulated the expression of* LPL* gene in both bone marrow- and gestational tissue-derived MSCs, its effects on* ADIPOQ* and* PPARγ* gene seem to be tissue specific. High glucose upregulated the expression of* ADIPOQ* only in CH-MSCs and UC-MSCs but not in BM-MSCs. In contrast, high glucose upregulated the expression of* PPARγ* only in BM-MSCs but not in gestational tissue-derived MSCs.

The differences in adipogenic gene expression might arise from two possibilities. Firstly, there are endogenous differences in the adipogenic differentiation capacity between bone marrow- and gestational tissue-derived MSCs. Our results showed that the upregulation of* ADIPOQ* and* PPARγ* genes in BM-MSCs cultured in adipogenic differentiation medium was much greater than those of gestational tissue-derived MSCs cultured under the same condition. These results suggest that BM-MSCs have higher adipogenic differentiation capacity than gestational tissue-derived MSCs. The upregulation of* ADIPOQ* gene in BM-MSCs cultured in adipogenic differentiation medium which is already 1200-fold higher than control even without glucose supplementation might obscure the positive effect of high glucose on the expression of this gene. In contrast to BM-MSCs, there is no upregulation of* ADIPOQ* gene in CH-MSCs and UC-MSCs cultured in adipogenic differentiation medium without glucose supplementation, so that the positive effect of high glucose on the expression of this gene became apparent. Secondly, it might also be possible that the adipocytes derived from gestational tissue-derived MSCs might be less mature than those derived from BM-MSCs; therefore, the expression levels of several adipogenic genes, such as* ADIPOQ*,* PPARγ*, and* LPL*, were much lower than those of BM-MSCs. This was supported by our observation that the adipocytes generated from gestational tissue-derived MSCs contain less amount of lipid droplets in comparison to the adipocytes generated from BM-MSCs.

High glucose might induce adipogenic differentiation of bone marrow- and gestational tissue-derived MSCs by increasing the production of reactive oxygen species (ROS) [11]. The increasing level of ROS has been reported to induce PKC signalling and lead to the adipogenic differentiation of both human adipose- and skeletal muscle-derived stem cells [11].

With regard to the osteogenic differentiation, we found that high glucose downregulated the expression of osteogenic gene* RUNX2* in CH-MSCs and UC-MSCs as well as* OSX* in CH-MSCs, PL-MSCs, and UC-MSCs on culture day 7. However, the downregulation of those genes could not be detected on culture day 14. We also did not find any difference in the levels of osteogenic differentiation of BM-MSCs, CH-MSCs, PL-MSCs, and UC-MSCs cultured under high glucose condition in comparison to their normal glucose controls.

Our results suggest that the effect of high glucose on the expression of osteogenic genes in MSCs is transient and its effect on the osteogenic differentiation of both BM-MSCs and gestational tissue-derived MSCs was not clearly demonstrated. These results are in contrast to the previous studies showing that high glucose suppressed the expression of osteogenic genes* RUNX2*,* OCN*,* COL1A1*, and* ON* in MG63 cells by activating cAMP/PKA/ERK pathway [9, 10]. This contrary is most likely arising from differences in biological properties between MG63 and gestational tissue-derived MSCs and the way they respond to high glucose.

## 5. Conclusion

We herein reported for the first time the effects of high glucose on the proliferation and adipogenic and osteogenic differentiation of several gestational tissue-derived MSCs including CH-MSCs, PL-MSCs, and UC-MSCs. Our result demonstrated that high glucose inhibits proliferation and enhances adipogenic differentiation of BM-MSCs, CH-MSCs, PL-MSCs, and UC-MSCs by upregulating the expressions of adipogenic genes* PPARγ*,* ADIPOQ*, and* LPL*. In contrast to adipogenic differentiation, high glucose did not affect osteogenic differentiation of all MSCs examined in this study. The knowledge gained from this study will increase our understanding on the mechanisms underlying the effects of high glucose on proliferation and adipogenic and osteogenic differentiation of MSCs and might lead to an improvement in the treatments of diabetes, obesity, and metabolic syndrome in the near future.

## Figures and Tables

**Table 1:** The sequences of primers for qRT-PCR.

Gene	Forward primer	Reverse primer
*ADIPOQ*	5′-CCTGGTGAGAAGGGTGAGAA-3′	5′-CAATCCCACACTGAATGCTG-3′
*GLUT4*	5′-CTTCGAGACAGCAGGGGTAG-3′	5′-ACAGTCATCAGGATGGCACA-3′
*LPL*	5′-TCAACTGGATGGAGGAGGAG-3′	5′-GGGGCTTCTGCATACTCAAA-3′
*PPARγ*	5′-GACCACTCCCACTCCTTTGA-3′	5′-AGGCTCCACTTTGATTGCAC-3′
*SREBP1C*	5′-TTCTCACCTCCCAGCTCTGT-3′	5′-GGAGGCTTCTTTGCTGTGAG-3′
*RUNX2*	5′-GACAGCCCCAACTTCCTGT-3′	5′-CCGGAGCTCAGCAGAATAAT-3′
*OCN*	5′-CTCACACTCCTCGCCCTATT-3′	5′-TCAGCCAACTCGTCACAGTC-3′
*OSX*	5′-TGCTTGAGGAGGAAGTTCAC-3′	5′-CTGCTTTGCCCAGAGTTGTT-3′
*GAPDH*	5′-CAATGACCCCTTCATTGACC-3′	5′-TTGATTTTGGAGGGATCTCG-3′

*ADIPOQ: *adiponectin*, GLUT4: *glucose transporter type 4*, LPL: *lipoprotein lipase*, PPARγ: *peroxisome proliferator-activated receptor gamma*, SREBP1C: *sterol regulatory element-binding protein 1C*, RUNX2: *runt-related transcription factor 2,* OCN: *osteocalcin*, OSX: *osterix, and* GAPDH: *glyceraldehyde 3-phosphate dehydrogenase.
